# Artificial intelligence, human cognition, and conscious supremacy

**DOI:** 10.3389/fpsyg.2024.1364714

**Published:** 2024-05-13

**Authors:** Ken Mogi

**Affiliations:** ^1^Sony Computer Science Laboratories, Shinagawa, Japan; ^2^Collective Intelligence Research Laboratory, The University of Tokyo, Meguro, Japan

**Keywords:** conscious supremacy, artificial intelligence, consciousness, large language model, computation

## Abstract

The computational significance of consciousness is an important and potentially more tractable research theme than the hard problem of consciousness, as one could look at the correlation of consciousness and computational capacities through, e.g., algorithmic or complexity analyses. In the literature, consciousness is defined as what it is like to be an agent (i.e., a human or a bat), with phenomenal properties, such as qualia, intentionality, and self-awareness. The absence of these properties would be termed “unconscious.” The recent success of large language models (LLMs), such as ChatGPT, has raised new questions about the computational significance of human conscious processing. Although instances from biological systems would typically suggest a robust correlation between intelligence and consciousness, certain states of consciousness seem to exist without manifest existence of intelligence. On the other hand, AI systems seem to exhibit intelligence without consciousness. These instances seem to suggest possible dissociations between consciousness and intelligence in natural and artificial systems. Here, I review some salient ideas about the computational significance of human conscious processes and identify several cognitive domains potentially unique to consciousness, such as flexible attention modulation, robust handling of new contexts, choice and decision making, cognition reflecting a wide spectrum of sensory information in an integrated manner, and finally embodied cognition, which might involve unconscious processes as well. Compared to such cognitive tasks, characterized by flexible and *ad hoc* judgments and choices, adequately acquired knowledge and skills are typically processed unconsciously in humans, consistent with the view that computation exhibited by LLMs, which are pretrained on a large dataset, could in principle be processed without consciousness, although conversations in humans are typically done consciously, with awareness of auditory qualia as well as the semantics of what are being said. I discuss the theoretically and practically important issue of separating computations, which need to be conducted consciously from those which could be done unconsciously, in areas, such as perception, language, and driving. I propose conscious supremacy as a concept analogous to quantum supremacy, which would help identify computations possibly unique to consciousness in biologically practical time and resource limits. I explore possible mechanisms supporting the hypothetical conscious supremacy. Finally, I discuss the relevance of issues covered here for AI alignment, where computations of AI and humans need to be aligned.

## 1 Introduction

Recently, large language models (LLMs) have made rapid progress based on the transformer (Vaswani et al., 2017) architecture, exhibiting many skills emulating but perhaps not matching human cognition, which were nonetheless once considered to be beyond the reach of machine intelligence, such as appropriate text generation based on a context, summarizing, searching under instructions, and optimization. With the advent of advanced AI systems such as ChatGPT (Sanderson, 2023), questions are arising regarding the computational significance, if any, of consciousness. Despite some claims that LLMs are either already or soon becoming conscious (Long, 2023), many regard these generative AI systems as doing computation unconsciously, thus forgoing possible ethical issues involved in AI abuse (Blauth et al., 2022). Generic models of consciousness would also suggest the LLMs to be unconscious as a default hypothesis, unless otherwise demonstrated, e.g., by convincing behavior suggesting the presence of consciousness to an external observer or a theoretical reasoning supported by an academic consensus. If LLMs can or come close to pass human-level cognition tests such as the false belief task in the theory of mind (Charman and Baron-Cohen, 1992; Baron-Cohen, 2000), the Turing test (Turing, 1950), and Winograd schema challenge (Sakaguchi et al., 2021) with their unconscious processing, what, if any, is the computational significance of consciousness?

Here, these abilities would not be necessary conditions for consciousness, as newborns are conscious without manifesting these abilities. The existence of these abilities would certainly be regarded as sufficient conditions for consciousness, in the generally accepted view of the human mind.

The theory of mind is related to the function of consciousness in the reportability and social context. The Turing test is tightly coupled with language, semantics in particular, and therefore closely related to consciousness. The Winograd schema challenge is crucial in understanding natural language, which is concerned with the nature of language here and now, locally, independent of the statistical properties dealt with in LLMs. The relation between functions exhibited by LLMs and consciousness is an interesting and timely question, especially when considering that natural language is typically processed when a human subject is conscious, except in the anecdotal and infrequent case of conversation in unconscious states, such as somniloquy (Reimão and Lefévre, 1980), hypnosis (Sarbin, 1997), and in a dream (Kilroe, 2016), which is a state distinctive from typical conscious or unconscious states. In an apparent contradiction to the conventional assumption about the necessity of consciousness in typical natural language exchanges, computations demonstrated by LLMs are considered to be done unconsciously. If conversations involving texts partially or totally generated by LLMs virtually pass the Turing test, without computations involving consciousness, what, if any, does consciousness do computationally?

Velmans (1991) analyzed the function of consciousness in cortical information processing, taking into account the role of focus of attention, concluding that it was not clear if consciousness was necessary for cognitive processes, such as perception, learning, and creativity. Velmans elaborated on the complexity of speech production, where the tongue may make as many as 12 adjustments of shape per second, so that “within 1 min of discourse as many as 10–15 thousand neuromuscular events occur” (Lenneberg, 1967). Based on these observations, Velmans suggested that speech production does not necessarily require consciousness. Such observations would necessitate a more nuanced consideration of the role of conscious and unconscious processes in language.

Apart from the conscious/unconscious divide, language occupies a central position in our understanding of consciousness. Velmans (2012) streamlined the foundations of consciousness studies, pointing out that the default position would be to reduce subjective experiences to objectively observable phenomena, such as brain function. On a more fundamental level, Velmans argued that language is associated with the dual-aspect nature of the psychophysical element of human experience, where language models the physical world only in incomplete ways, limited by the capacities of our senses. The central role of language in our understanding of the world, including consciousness, should be kept in mind when discussing artificial reproductions of language, including, but not limited to, the LLMs.

Many regard the problem of consciousness as primarily in the phenomenological domain, concerned with what is experienced by a subject when he or she is conscious, e.g., properties such as qualia, intentionality, and self-awareness as opposed to physical or functional descriptions of the brain function. There are experimental and theoretical approaches tackling the cognitive implications of consciousness based on ideas, such as neural correlates of consciousness (NCC, Crick and Koch, 1998; Koch et al., 2016), global workspace theory (Baars, 1997, 2005), integrated information theory (Tononi et al., 2016), and free-energy principle (Friston, 2010).

Wiese and Friston (2021) discussed the relevance of the free-energy principle as a constraint for the computational correlates of consciousness (CCC), stressing the importance of neural dynamics, not states. In their framework, trajectories rather than states are mapped to conscious experiences. They propose CCC as a more general concept than the neural correlates of consciousness (NCC), discussing the nature of the correlates as necessary, sufficient, or both conditions for consciousness.

Some, somewhat controversially, consider quantum effects as essential in explaining the nature of consciousness (Hameroff, 1998; Woolf and Hameroff, 2001). Although there have been significant advances made, explaining the hard problem of consciousness (Chalmers, 1995) from such theoretical approaches remains hypothetical at best, even if not cognitively closed (McGinn, 1994), and a scientific consensus has not been reached yet. There are also arguments that hold that the hard problem is not necessarily essential for the study of consciousness. Seth (2021) argued that if we pursue the real problem of accounting for properties of consciousness in terms of biological mechanisms, the hard problem will turn out to be less important.

Given the difficulty in studying the phenomenological aspects of consciousness, with the advancement in artificial intelligence (AI), there is now a unique opportunity to study the nature of consciousness by approaching it from its computational significance. As artificial intelligence systems, such as LLMs, are reproducing and even surpassing human information processing capabilities, the identification of computational elements possibly unique to consciousness is coming under more focused analysis.

At present, it is difficult to give a precise definition of what computations unique to consciousness are. What follows are tentative descriptions adopted in this paper. From the objective point of view, neural computation correlating with consciousness would typically involve large areas of the brain processing information in coherent and integrated parallel manners, while sensory qualia represent the result of complex processing in compressed forms, as in color constancy (Foster, 2011). Unconscious computation, on the other hand, does not meet these criteria. From the subjective point of view, conscious computation would be accompanied by such properties as qualia, intentionality, and self-consciousness. Unconscious computations do not cause these aspects of experience to emerge.

Artificial intelligence is an umbrella term, and its specific capabilities depend on parameters and configurations of system makeup and dynamics. For now, we would assume that AI systems referred to here are realized on classical computers. AI systems constructed on quantum computers might exhibit broader ranges of computational capabilities, possibly exhibiting quantum supremacy (Arute et al., 2019), which describes the abilities of quantum computers to solve problems any classical computer could not solve in any practical time. Quantum supremacy is not a claim that quantum computers would be able to execute computations beyond what universal Turing machines (Turing, 1936) are capable of. It is rather a claim that quantum computers can, under the circumstances, execute computations that could, in principle, be done by classical computers, but not within any practical period considering the physical time typically available to humans.

Similarly, conscious supremacy can be defined as domains of computation that can be conducted by conscious processes but cannot be executed by systems lacking consciousness in any practical time. Since the science of consciousness has not yet developed to reach the same level as quantum mechanics, it is difficult to give a precise definition of what conscious supremacy is at present. What follows is a tentative definition adopted in this article. Out of all the computations done in the neural networks in the brain, conscious supremacy refers to those areas of computation accompanied by consciousness, which are done in efficient and integrated ways compared to unconscious computation. Given the limits of resources available in the brain, computations executed in conscious supremacy would be, in a practical sense, impossible to execute by unconscious computation in any meaningful biological time. However, in principle, they could be done. Thus, there are no distinctions between computations belonging to conscious supremacy and other domains in terms of computability in principle. The practical impossibility of non-conscious systems to execute computations belonging to conscious supremacy would have been one of the adaptive values of consciousness in evolution.

The relationship between quantum supremacy and conscious supremacy will be discussed later.

As of now, quantum supremacy remains controversial (McCormick, 2022). The merit of introducing the perhaps equally debatable concept of conscious supremacy is that we can hope to streamline aspects of computation conducted by conscious and unconscious processes.

Abilities to play board games, such as chess, shogi, and go, are no longer considered to be unique to human cognition after AI systems, such as Deep Blue (Campbell et al., 2002) and AlphaZero (Schrittwieser et al., 2020), defeated human champions. After the success of LLMs in executing a large part of natural language tasks, cognitive abilities once considered unique to humans, e.g., the theory of mind, Turing test, and Winograd schema challenge, might not be considered to be verifications of the ability of artificial intelligence systems to perform cognitive tasks on par with humans. It should be noted that the attribution of the theory of mind to LLMs remains controversial (Aru et al., 2023), and the exact nature of cognitive functions related to natural language, if any, in LLMs is an open question. However, it does seem legitimate to start considering the exclusion of certain computations from the set of those unique to consciousness based on computational evidence. While such exclusion might reflect cognitive biases on the part of humans to raise the bar unfavorably for AI systems, in an effort to solve cognitive dissonance (Aronson, 1969) about the relative superiorities of AI and humans, such considerations could serve as a filter to fine-tune domains of cognitive tasks uniquely executed by human cognition, conscious, and unconscious.

**Figure 1:**
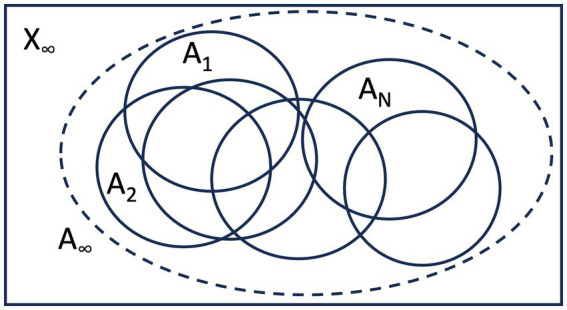
The analysis of AI capabilities would help focus the computational domain unique to consciousness (X), which can be defined in terms of instances of AI systems. As the number of AI systems increases, computations unique to consciousness will be more finely defined.

As artificial intelligence systems based on deep learning and other approaches advance in their abilities, tasks considered to be uniquely human would gradually diminish in the spectrum of functionalities. Specifically, the set X of computations considered unique to humans would be the complement of the union of the set of computations executed by artificial intelligence systems A_1_, A_2_, …, A_N_ under consideration. Namely, X = A^c^, where A = A_1_UA_2_U… UA_N_ (Figure 1), where the whole set represents the space of possible computations conducted by humans. As the number of artificial intelligence systems increases, the uniquely human domain of computation would ultimately become X_∞_ = A_∞_^c^, where A_∞_ = lim_N- > ∞_A_1_UA_2_U… UA_N_.

Needless to say, such an argument is conceptual in nature, as it is difficult to draw a clear line between what could and could not be done by artificial intelligence systems at present. Among computations unique to humans, some would be executed consciously, while some might be a combination of conscious and unconscious computation, involving processes which lie either inside or outside the neural correlates of consciousness (Crick and Koch, 1998; Koch et al., 2016). Theoretically, there could also be computations unique to humans executed unconsciously, although not of central interest in the context adopted here.

Penrose suggested that consciousness is correlated with the quantum mechanical effect, possibly involving quantum gravity (Penrose, 1996). Penrose went on to collaborate with Stuart Hameroff. Penrose and Hameroff together suggested, in a series of papers (Hameroff and Penrose, 1996; Hameroff and Penrose, 2014), that quantum mechanical processes in microtubules were involved in conscious processes, which went beyond the algorithmic capabilities of computability for the classical computer. Specifically, it was postulated that a process named “Orchestrated objective reduction” (Orch OR) was responsible for the generation of proto-consciousness in microtubules, a hypothesis independent from conventional arguments on quantum computing. One of the criticisms directed to such quantum models of consciousness was based on the fact that temperatures in biological systems are typically too high for quantum coherence or entanglement to be effective (Tegmark, 2000).

## 2 Possibilities and limits of artificial intelligence systems

Artificial General Intelligence (AGI; Goertzel, 2014) is purported to execute all tasks carried out by a typical human brain and beyond. Proposed tasks to be executed by AGI include the Turing test, coffee making or Wozniak test (Adams et al., 2012), college enrollment test (Goertzel, 2014), employment test (Scott et al., 2022), and the discovery of new scientific knowledge (Kitano, 2016).

In identifying possible areas for uniquely human cognition and potential candidates for conscious supremacy, it is useful to discuss systemic potentials and limits of artificial intelligence, which are currently apparent.

Some LLMs have started to show sparks of general intelligence (Bubeck et al., 2023) beyond abilities for linguistic processing. Such a potential might be explained by the inherent functions of language. The lexical hypothesis (Crowne, 2007) states that important concepts in fields, such as personality study and general philosophy, would be expressible by everyday language. The ability of natural language to represent and analyze a wide range of information in the environment is consistent with the perceived general ability of LLMs to represent various truths about this world, without necessarily being conscious, thus suggesting the central importance of representation in the analysis of intelligence.

What is meant by representation is a potentially controversial issue. In the conventional sense of psychology and philosophy of mind, a representation refers to the internal state that corresponds to an external reality (Marr, 1982). In the constructivist approach, representation would be an active construct of an agent’s knowledge, not necessarily requiring an external reality as a prior (Von Glasersfeld, 1987). Representations in artificial intelligence systems would be somewhere in between, taking inspiration from various lines of theoretical approaches.

One of the problems with LLMs, such as ChatGPT, is the occurrence of hallucination (Ji et al., 2023) and the tendency to produce sentences inconsistent with accepted facts, a term criticized by some researchers as an instance of anthropomorphism. Although humans also suffer from similar misconceptions, subjects typically are able to make confident judgments about their own statements (Yeung and Summerfield, 2012), while methods for establishing similar capabilities in artificial intelligence systems have not been established. Regarding consciousness, metacognitive processes associated with consciousness (Nelson, 1996) might help rectify potential errors in human cognition.

Behaviorist ways of thinking (Araiba, 2019) suggest that human thoughts are ultimately represented in terms of bodily movements. No matter how well developed an intelligent agent might be, manifestations of its functionality would ultimately be found in its objective courses of action in the physical space. From this perspective, the intelligence of an agent would be judged in terms of its external behavior, an idea in AI research sometimes called instrumental convergence (Bostrom, 2012).

The possibilities and limits of artificial intelligence systems would be tangibly assessed through analysis of behavior. In voluntary movement, evidence suggests that consciousness is involved in vetoing a particular action (free won’t) when it is judged to be inappropriate within a particular context (Libet, 1999).

Thus, from robust handling of linguistic information to streamlining of external behavior, metacognitive monitoring and control would be central in identifying and rectifying limits of artificial intelligence systems, a view consistent with the idea that metacognition plays an essential role in consciousness (Nelson, 1996).

## 3 Computations possibly unique to conscious processing

As of now, the eventual range of computational capabilities of artificial intelligence is unclear. Employing cognitive arguments based on the observation of what subset of computation is typically done consciously, in addition to insights on the limits of artificial intelligence, would help narrow down possible consciousness-specific tasks. In that process, the division of labor between conscious and unconscious processes could be made, as we thus outline heterogeneous aspects of cognition.

Acquiring new skills or making decisions in novel contexts would typically require the involvement of conscious processing, while the execution of acquired skills would proceed largely unconsciously (Solomon, 1911; Lisman and Sternberg, 2013) in terms of the accompanying phenomenological properties, such as qualia, intentionality, and attention. Any cognitive task, when it needs to integrate information analyzed across many different regions in the brain, typically requires consciousness, reflecting the global nature of consciousness in terms of cortical regions involved (Baars, 2005). The autonomous execution of familiar tasks would involve a different set of neural networks compared to the minimum set of neural activities (neural correlates, Koch et al., 2016) required for the sustaining of consciousness.

It is interesting to note here that some self-learning unsupervised artificial intelligence systems seem to possess abilities to acquire new skills and make decisions in novel contexts (Silver et al., 2017; Schrittwieser et al., 2020). As the ability of artificial intelligence systems approaches the level purported for AGI (Goertzel, 2014), the possibility of the emergence of consciousness might have to be considered.

The global neural workspace (GNW) theory (Dehaene et al., 1998; Mashour et al., 2020) addresses how the neural networks in the brain support a dynamic network where relevant information can be assessed by local networks, eventually giving rise to consciousness. The multimodal nature of the GNW theory has inspired various theoretical works, including those related to deep learning networks (LeCun et al., 2015; Bengio, 2017).

In evolution, one of the advantages of information processing involving consciousness might have been decision-making reflecting a multitude of sensory inputs. Multimodal perception typically subserves such a decision-making process. Since the science of decision-making is an integral part of AI alignment (Yudkowsky, 2015), the difference between conscious and unconscious, as well as human and AI decision-making processes, would shed much light on the parameters of systems supporting the nature of conscious computation.

Technological issues surrounding self-driving cars (Badue et al., 2021) have emerged as one of the most important research themes today, both from theoretical and practical standpoints. Driving cars involves a series of judgments, choices, and actions based on multimodal sensory information. Judgments on how to drive a vehicle often must be done within limited time windows in *ad hoc* situations, affected by the unpredictability of other human drivers, if any, and there are still challenges toward realizing fully self-driving vehicles (Kosuru and Venkitaraman, 2023). Moral dilemmas involved in driving judgments require sorting out situations concerned with conflicting choices for safety, known collectively as the trolley problem (Thomson, 1985), which is often intractable even when presented with clear alternative schemes (Awad et al., 2018). In real-life situations, there would be perceptual and cognitive ambiguities about, for example, whether you can really save five people by sacrificing one. In the face of such difficulties, fully self-driving cars without conscious human interventions might turn out to be impossible (Shladover, 2016).

The language is a series of micro-decisions, in that words must be selected, depending on the context, as follow-up sequences on what has been already expressed. The apparent success of LLMs in reproducing salient features of embedded knowledge in the language (Singhal et al., 2023) is impressive. However, it might still fall short of executing situated or embodied choice of words, as required, for example, in the college enrollment and employment tests. A linguistic generative AI might nominally pass the Turing test in artificial and limited situations. However, when an AI system implemented in a robot interacts with a human in real-life situations, there might be a perceived uncanny valley (Mori, 2012) linguistically, where negative emotions, such as uneasiness and repulsion, might be hypothetically induced in a human subject as the performance comes nearer to the human level.

## 4 Possible mechanisms for conscious supremacy

It is possible that there are computations uniquely executed by conscious processes, and there could be some similarities between conscious and quantum computations, independent of whether consciousness actually involves quantum processes in the brain. There could be similarities between postulated quantum supremacy and conscious supremacy, without underlying common mechanisms being necessarily implicated. It is worth noting here that just as it is in principle possible to simulate quantum computing on classical computers, it might be possible to simulate conscious computing, regardless of its nature, on classical computers, e.g., in terms of connectionist models representing neural networks in the brain.

There are several algorithms that demonstrate the superiority of quantum computing. For example, Schor’s algorithm (Shor, 1994) can find prime factors of large numbers efficiently. Given a large number N, Shor’s algorithm for finding prime factors can run in polynomial time in terms of N, compared to sub-exponential time on optimal algorithms for a classical computer.

**Figure 2:**
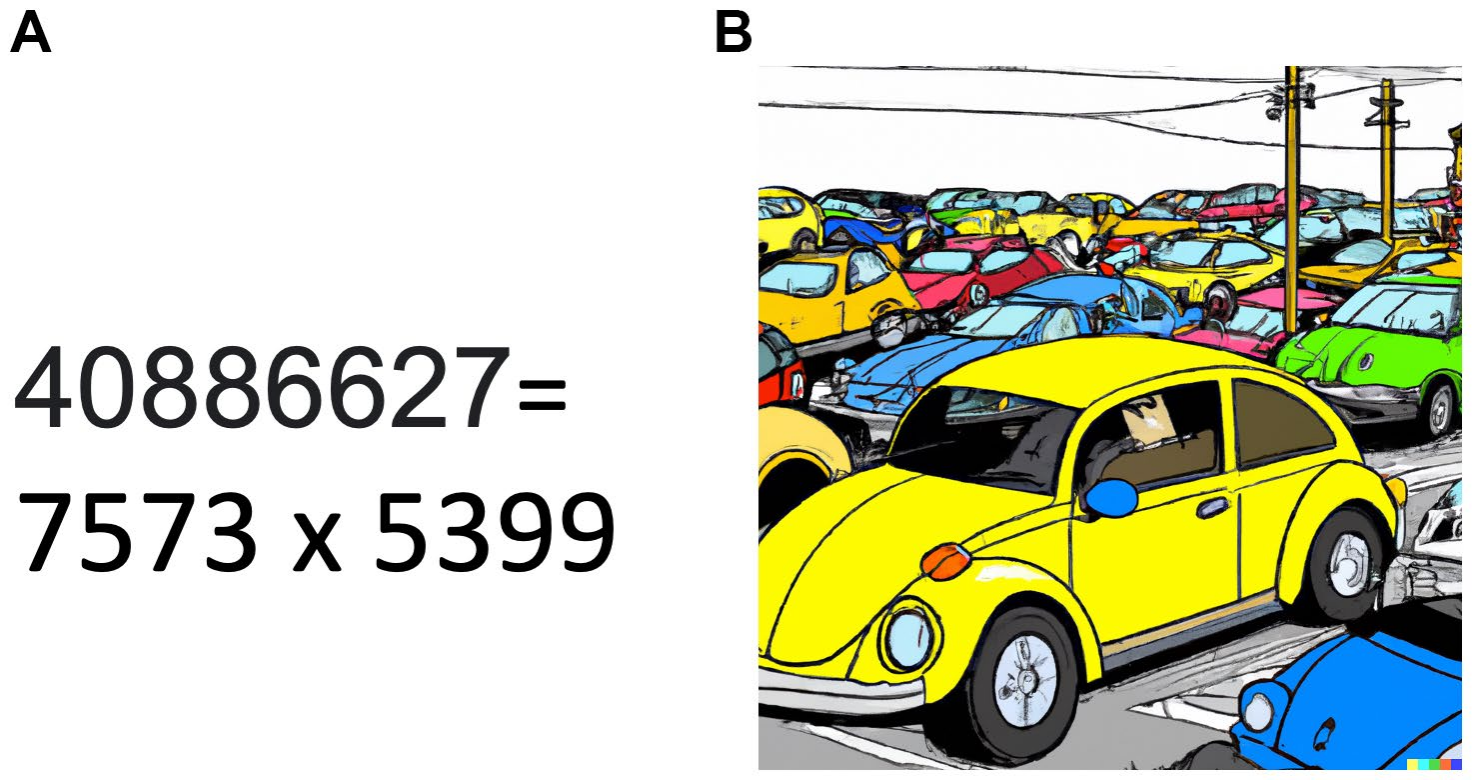
Analogy between finding prime factors and integration of visual features. **(A)** Finding prime factors for a large number becomes increasingly difficult for classical computers. Quantum computing employing Shor’s algorithm provides an efficient method for factoring large natural numbers. **(B)** Sorting out combinatorial explosion in the integration of visual features represented in distributed neural networks in the brain is a still unresolved challenge known as the binding problem. The picture was generated by Dall-E (Open AI) with the prompt: A yellow Volkswagen Beetle car surrounded by cars of different shapes and colors seen from a distance in manga style.

In conscious visual perception, the binding problem (Feldman, 2012) questions how the brain integrates visual features, such as colors and forms, into coherent conscious percepts. The challenge of combinatorial explosion (Treisman, 1999), in which all possible combinations of features, such as the yellow (color) Volkswagen Beetle car (form), must be dealt with, becomes essential there. Given the fact that forms (Logothetis et al., 1995) and colors (Zeki and Marini, 1998) are represented by distributed circuits in the brain, sorting through the possible combinations of forms and colors has similarities with the factoring problem addressed by Shor’s algorithm (Figure 2).

In quantum computing (Deutsch, 1985; Feynman, 1985), quantum superposition and entanglement are ingeniously employed to conduct algorithms effectively impossible for classical computers to execute in realistic time frames. In a quantum computing process, decoherence would introduce noise, and in order to execute on a large scale, a process called quantum error correction (QEC; Cai and Ma, 2021) is essential.

In conscious computing discussed here, similar mechanisms might be at play. For example, the contrast between the noisy neural firings and the apparently Platonic phenomenology of qualia suggests a process in which the variabilities due to noise in neural firings are rectified, named here conscious error correction (CEC). At present, the plausibility or the details of such an error-rectifying scheme is not clear. The possible relationships (if any) between QEC and CEC remain speculative at best at the moment. Despite these reservations, the involvement of error-correcting mechanisms in consciously conducted computation would be a line of thought worth investigating.

## 5 Implications for AI alignment

As artificial intelligence systems make progress, it is becoming important to align them with humans, an area called AI alignment (Russell and Norvig, 2021).

The elucidation of computations uniquely executed by consciousness and the possible existence of conscious supremacy, i.e., computations specifically and uniquely executed by neural processes correlating with consciousness, would put a constraint on AI alignment schemes.

Specifically, it would be an efficient alignment strategy to develop AI systems with capabilities other than uniquely conscious computations, while leaving computation involving conscious supremacy to humans.

It is interesting to consider the implications of such divisions of labor between AIs and humans for AI safety (Zhang et al., 2021). It would be impractical to require AI systems to carry out tasks better left to humans. Expecting AIs to execute tasks belonging to conscious supremacy would significantly disrupt AI safety.

Eliezer Yudkowsky’s conceptualization of Friendly AI (Yudkowsky, 2008) is based on the importance of updating the system in accordance with humans (Russell and Norvig, 2021). Reinforcement learning from human feedback (RLHF; Stiennon et al., 2020), a technique often used in the development of artificial intelligence systems, can be considered to be an instance of developing Friendly AI and an attempt at the division of labor between conscious (human) and unconscious (AI) computations.

Alignment of AIs with humans, in the context of AI safety in particular, would depend on an effective division of labor between cognition unique to humans centered on conscious supremacy and computation conducted by computers, in a way similar to the interaction between conscious and unconscious processes in the human brain. In this context, artificial intelligence systems can be regarded as extensions of unconscious processes in the brain. Insights on cortical plasticities from tool use (Iriki et al., 1996) could provide relevant frameworks for discussion. It is important to note that limiting the functions of artificial intelligence systems to non-conscious operations does not necessarily guarantee robust alignment. Alignment would also depend on parameters that are dependent on the developers and stakeholders in the ecosystem of artificial intelligence. It would be important to discuss various aspects concerning alignment, including those put forward here.

Finally, the development of artificial consciousness (Chrisley, 2008), whether theoretically or practically feasible or not, might not be an effective strategy for AI alignment. From the point of view of the division of labor, computational domains belonging to conscious supremacy would be better left to humans. Artificial intelligence systems would do a better job of alignment by trying to augment computations unique to consciousness, which are to be reasonably executed by humans, rather than by replacing them from scratch.

## 6 Discussion

I have addressed here the possibility of characterizing conscious processes from a computational point of view. The development of artificial intelligence systems provides unique opportunities to explore and focus more deeply on computational processes unique to consciousness.

At present, it is not clear whether consciousness would eventually emerge from present lines of research and development in artificial intelligence. It would be useful to start from the null hypothesis of the non-existence of consciousness in artificial intelligence systems. We would then be able to narrow down what consciousness uniquely computes.

I have proposed the concept of conscious supremacy. Although this is speculative at present, it would be useful to think in terms of computational contexts apart from the hard problem of the phenomenology of consciousness. The presence of conscious supremacy would be connected to the advantages the emergence of consciousness has provided in the history of evolution. Elucidating the nature of conscious supremacy would help decipher elements involved in consciousness, whether it is ultimately coupled with quantum processes or not.

The value of arguments presented in this paper is limited, as it has not yet specifically identified computations unique to consciousness. The efforts to characterize computations unique to consciousness in terms of conscious supremacy presented here would hopefully help streamline discussions on this issue, although, needless to say, much work remains to be done.

## Data availability statement

The original contributions presented in the study are included in the article/supplementary material, further inquiries can be directed to the corresponding author.

## Author contributions

KM: Conceptualization, Data curation, Formal analysis, Funding acquisition, Investigation, Methodology, Project administration, Resources, Software, Supervision, Validation, Visualization, Writing – original draft, Writing – review & editing.

## References

[ref1] Adams S. Arel I. Bach J. Coop R. Furlan R. Goertzel B.. (2012). Mapping the landscape of human-level artificial general intelligence. AI Mag. 33, 25–41. doi: 10.1609/aimag.v33i1.2322

[ref2] Araiba S. (2019). Current diversification of behaviorism. Perspect. Beha. Sci. 43, 157–175. doi: 10.1007/s40614-019-00207-0, PMID: 32440649 PMC7198672

[ref3] Aronson E. (1969). “The theory of cognitive dissonance: a current perspective” in Advances in experimental social psychology, vol. 4 (Academic Press), 1–34.

[ref4] Aru J. Labash A. Corcoll O. Vicente R. (2023). Mind the gap: challenges of deep learning approaches to theory of mind. Artif. Intell. Rev. 56, 9141–9156. doi: 10.1007/s10462-023-10401-x

[ref5] Arute F. Arya K. Babbush R. Bacon D. Bardin J. C. Barends R.. (2019). Quantum supremacy using a programmable superconducting processor. Nature 574, 505–510. doi: 10.1038/s41586-019-1666-5, PMID: 31645734

[ref6] Awad E. Dsouza S. Kim R. Schulz J. Henrich J. Shariff A.. (2018). The moral machine experiment. Nature 563, 59–64. doi: 10.1038/s41586-018-0637-6 30356211

[ref7] Baars B. J. (1997). In the theatre of consciousness. Global workspace theory, a rigorous scientific theory of consciousness. J. Conscious. Stud. 4, 292–309.

[ref8] Baars B. J. (2005). Global workspace theory of consciousness: toward a cognitive neuroscience of human experience. Prog. Brain Res. 150, 45–53. doi: 10.1016/S0079-6123(05)50004-9 16186014

[ref9] Badue C. Guidolini R. Carneiro R. V. Azevedo P. Cardoso V. B. Forechi A.. (2021). Self-driving cars: a survey. Expert Syst. Appl. 165:113816. doi: 10.1016/j.eswa.2020.113816

[ref10] Baron-Cohen S. (2000). Theory of mind and autism: a review. Int. Rev. Res. Mental Retardat. 23, 169–184. doi: 10.1016/S0074-7750(00)80010-5

[ref11] Beckman D. Chari A. N. Devabhaktuni S. Preskill J. (1996). “Efficient networks for quantum factoring” (PDF). Phys. Rev. A 54, 1034–1063. doi: 10.1103/PhysRevA.54.1034, PMID: 9913575

[ref12] Bengio Y. (2017). The consciousness prior. arXiv:1709.08568. doi: 10.48550/arXiv.1709.08568

[ref13] Benioff P. (1980). The computer as a physical system: a microscopic quantum mechanical Hamiltonian model of computers as represented by Turing machines. J. Stat. Phys. 22, 563–591. doi: 10.1007/BF01011339

[ref14] Blauth T. F. Gstrein O. J. Zwitter A. (2022). Artificial intelligence crime: an overview of malicious use and abuse of AI. IEEE Access 10, 77110–77122. doi: 10.1109/ACCESS.2022.3191790

[ref15] Bostrom N. (2012). The superintelligent will: motivation and instrumental rationality in advanced artificial agents. Mind. Mach. 22, 71–85. doi: 10.1007/s11023-012-9281-3

[ref16] Bray D. (1995). Protein molecules as computational elements in living cells. Nature 376, 307–312. doi: 10.1038/376307a0 7630396

[ref17] Bubeck S. Chandrasekaran V. Eldan R. Gehrke J. Horvitz E. Kamar E.. (2023). Sparks of artificial general intelligence: early experiments with gpt-4. arXiv preprint 2303.12712. doi: 10.48550/arXiv.2303.12712

[ref18] Cai W. Ma Y. (2021). Bosonic quantum error correction codes in superconducting quantum circuits. Fundamental Res. 1, 50–67. doi: 10.1016/j.fmre.2020.12.006

[ref19] Campbell M. Hoane A. J. Jr. Hsu F. H. (2002). Deep Blue. Artif. Intell. 134, 57–83. doi: 10.1016/S0004-3702(01)00129-1

[ref1001] Chalmers D. (1995). Facing up to the problem of consciousness. J. Conscious. 2, 200–219.

[ref20] Charman T. Baron-Cohen S. (1992). Understanding drawings and beliefs: a further test of the metarepresentation theory of autism: a research note. J. Child Psychol. Psychiatry 33, 1105–1112. doi: 10.1111/j.1469-7610.1992.tb00929.x, PMID: 1400691

[ref21] Chrisley R. (2008). Philosophical foundations of artificial consciousness. Artif. Intell. Med. 44, 119–137. doi: 10.1016/j.artmed.2008.07.011, PMID: 18818062

[ref22] Crick F. Koch C. (1998). Consciousness and neuroscience. Cereb. Cortex 8, 97–107. doi: 10.1093/cercor/8.2.97 9542889

[ref23] Crowne D. P. (2007). Personality theory. Oxford: Oxford University Press.

[ref24] Dehaene S. Kerszberg M. Changeux J. P. (1998). A neuronal model of a global workspace in effortful cognitive tasks. Proc. Natl. Acad. Sci. 95, 14529–14534. doi: 10.1073/pnas.95.24.14529, PMID: 9826734 PMC24407

[ref25] Deutsch D. (1985). Quantum theory, the church–Turing principle and the universal quantum computer. Proceedings of the Royal Society of London. A. Math. Phys. Sci. 400, 97–117.

[ref26] Feldman J. (2012). The neural binding problem. Cogn. Neurodyn. 7, 1–11. doi: 10.1007/s11571-012-9219-8, PMID: 24427186 PMC3538094

[ref27] Feynman R. P. (1985). Quantum mechanical computers. Optics News 11, 11–20. doi: 10.1364/ON.11.2.000011

[ref1002] Foster D. H. (2011). Color constancy. Vis. Res. 51, 674–700. 20849875 10.1016/j.visres.2010.09.006

[ref28] Friston K. (2010). The free-energy principle: a unified brain theory? Nat. Rev. Neurosci. 11, 127–138. doi: 10.1038/nrn2787 20068583

[ref29] Goertzel B. (2014). Artificial general intelligence: concept, state of the art, and future prospects. J. Artif. Gen. Intell. 5, 1–48. doi: 10.2478/jagi-2014-0001

[ref30] Hameroff S. (1998). Quantum computation in brain microtubules? The Penrose–Hameroff ‘Orch OR ‘model of consciousness. Philos. Trans. R. Soc. London, Ser. A 356, 1869–1896

[ref31] Hameroff S. R. Penrose R. (1996). Conscious events as orchestrated space-time selections. J. Conscious. Stud. 3, 36–53.

[ref32] Hameroff S. Penrose R. (2014). Consciousness in the universe: a review of the ‘Orch OR’theory. Phys Life Rev 11, 39–78. doi: 10.1016/j.plrev.2013.08.002 24070914

[ref33] Iriki A. Tanaka M. Iwamura Y. (1996). Coding of modified body schema during tool use by macaque postcentral neurones. Neuroreport 7, 2325–2330. doi: 10.1097/00001756-199610020-00010, PMID: 8951846

[ref34] Ji Z. Lee N. Frieske R. Yu T. Su D. Xu Y.. (2023). Survey of hallucination in natural language generation. ACM Comput. Surv. 55, 1–38. doi: 10.1145/3571730

[ref35] Kilroe P. A. (2016). Reflections on the study of dream speech. Dreaming 26, 142–157. doi: 10.1037/drm0000016

[ref36] Kitano H. (2016). Artificial intelligence to win the nobel prize and beyond: creating the engine for scientific discovery. AI Mag. 37, 39–49. doi: 10.1609/aimag.v37i1.2642

[ref37] Koch C. Massimini M. Boly M. Tononi G. (2016). Neural correlates of consciousness: progress and problems. Nat. Rev. Neurosci. 17, 307–321. doi: 10.1038/nrn.2016.22, PMID: 27094080

[ref38] Kosuru V. S. R. Venkitaraman A. K. (2023). Advancements and challenges in achieving fully autonomous self-driving vehicles. World J. Adv. Res. Rev. 18, 161–167. doi: 10.30574/wjarr.2023.18.1.0568

[ref39] Lau H. Rosenthal D. (2011). Empirical support for higher-order theories of conscious awareness. Trends Cogn. Sci. 15, 365–373. doi: 10.1016/j.tics.2011.05.009, PMID: 21737339

[ref40] LeCun Y. Bengio Y. Hinton G. (2015). Deep learning. Nature 521, 436–444. doi: 10.1038/nature14539 26017442

[ref41] Lenneberg E. H. (1967). Biological foundations of language, vol. 2. New York: Wiley, 59–67.

[ref42] Libet B. (1999). Do we have free will? J. Conscious. Stud. 6, 47–57.

[ref43] Lisman J. Sternberg E. J. (2013). Habit and nonhabit systems for unconscious and conscious behavior: implications for multitasking. J. Cogn. Neurosci. 25, 273–283. doi: 10.1162/jocn_a_00319, PMID: 23163411

[ref44] Logothetis N. K. Pauls J. Poggio T. (1995). Shape representation in the inferior temporal cortex of monkeys. Curr. Biol. 5, 552–563. doi: 10.1016/S0960-9822(95)00108-4 7583105

[ref45] Long R. (2023). Introspective capabilities in large language models. J. Conscious. Stud. 30, 143–153. doi: 10.53765/20512201.30.9.143

[ref46] Marr D. (1982). Vision: A computational investigation into the human representation and processing of visual information. New York: W. H. Freeman and Company.

[ref47] Mashour G. A. Roelfsema P. Changeux J. P. Dehaene S. (2020). Conscious processing and the global neuronal workspace hypothesis. Neuron 105, 776–798. doi: 10.1016/j.neuron.2020.01.026, PMID: 32135090 PMC8770991

[ref48] McCormick K. (2022). Race not over between classical and quantum computers. Physics 15:19. doi: 10.1103/Physics.15.19

[ref49] McGinn C. (1994). The problem of philosophy. Philos. Stud. 76, 133–156. doi: 10.1007/BF00989821

[ref50] Mori M. (2012). The uncanny valley. IEEE Robot. Automat. 19, 98–100. doi: 10.1109/MRA.2012.2192811

[ref51] Nelson T. O. (1996). Consciousness and metacognition. Am. Psychol. 51, 102–116. doi: 10.1037/0003-066X.51.2.102

[ref52] Penrose R. (1996). On gravity’s role in quantum state reduction. Gen. Relativ. Gravit. 28, 581–600. doi: 10.1007/BF02105068

[ref53] Reimão R. N. Lefévre A. B. (1980). Prevalence of sleep-talking in childhood. Brain Dev. 2, 353–357. doi: 10.1016/S0387-7604(80)80047-7 7224091

[ref54] Russell S. J. Norvig P. (2021). Artificial intelligence: A modern approach. 4th Edn. London: Pearson.

[ref55] Sakaguchi K. Bras R. L. Bhagavatula C. Choi Y. (2021). Winogrande: an adversarial winograd schema challenge at scale. Commun. ACM 64, 99–106. doi: 10.1145/3474381

[ref56] Sanderson K. (2023). GPT-4 is here: what scientists think. Nature 615:773. doi: 10.1038/d41586-023-00816-5, PMID: 36928404

[ref57] Sarbin T. R. (1997). Hypnosis as a conversation:‘believed-in imaginings’ revisited. Contemp. Hypn. 14, 203–215. doi: 10.1002/ch.105

[ref58] Schrittwieser J. Antonoglou I. Hubert T. Simonyan K. Sifre L. Schmitt S.. (2020). Mastering atari, go, chess and shogi by planning with a learned model. Nature 588, 604–609. doi: 10.1038/s41586-020-03051-4, PMID: 33361790

[ref59] Scott A. C. Solórzano J. R. Moyer J. D. Hughes B. B. (2022). The future of artificial intelligence. Int. J. Artif. Intell. Mach. Learn. 2, 1–37. doi: 10.51483/IJAIML.2.1.2022.1-37

[ref60] Seth A. (2021). Being you: A new science of consciousness. New York: Penguin.

[ref61] Shladover S. E. (2016). The truth about “self-driving” cars. Sci. Am. 314, 52–57. doi: 10.1038/scientificamerican0616-52, PMID: 27196843

[ref62] Shor P. W. (1994). “Algorithms for quantum computation: discrete logarithms and factoring” in Proceedings 35th annual symposium on foundations of computer science (Washington, DC: IEEE Computer Society Press), 124–134.

[ref63] Silver D. Schrittwieser J. Simonyan K. Antonoglou I. Huang A. Guez A.. (2017). Mastering the game of go without human knowledge. Nature 550, 354–359. doi: 10.1038/nature24270, PMID: 29052630

[ref64] Singhal K. Azizi S. Tu T. Mahdavi S. S. Wei J. Chung H. W.. (2023). Large language models encode clinical knowledge. Nature 620, 172–180. doi: 10.1038/s41586-023-06291-2, PMID: 37438534 PMC10396962

[ref65] Solomon J. (1911). The philosophy of Bergson. Mind XX, 15–40. doi: 10.1093/mind/XX.77.15

[ref66] Stiennon N. Ouyang L. Wu J. Ziegler D. Lowe R. Voss C.. (2020). Learning to summarize with human feedback. Adv. Neural Inf. Proces. Syst. 33, 3008–3021. doi: 10.48550/arXiv.2009.01325

[ref67] Tegmark M. (2000). Importance of quantum decoherence in brain processes. Phys. Rev. E 61, 4194–4206. doi: 10.1103/PhysRevE.61.4194, PMID: 11088215

[ref68] Thomson J. J. (1985). The trolley problem. Yale Law J. 94, 1395–1415. doi: 10.2307/796133

[ref69] Tononi G. Boly M. Massimini M. Koch C. (2016). Integrated information theory: from consciousness to its physical substrate. Nat. Rev. Neurosci. 17, 450–461. doi: 10.1038/nrn.2016.44, PMID: 27225071

[ref70] Treisman A. (1999). Solutions to the binding problem: progress through controversy and convergence. Neuron 24, 105–125. doi: 10.1016/S0896-6273(00)80826-0, PMID: 10677031

[ref71] Turing A. (1936). On computable numbers, with an application to the Entscheidungsproblem. J. Math 58, 345–363.

[ref72] Turing A. (1950). Computing machinery and intelligence, mind. LIX LIX, 433–460. doi: 10.1093/mind/LIX.236.433

[ref73] Vaswani A. Shazeer N. Parmar N. Uszkoreit J. Jones L. Gomez A. N.. (2017). Attention is all you need. Adv. Neural Inf. Proces. Syst. 30, 6000–6010. doi: 10.48550/arXiv.1706.03762

[ref74] Velmans M. (1991). Is human information processing conscious? Behav. Brain Sci. 14, 651–669. doi: 10.1017/S0140525X00071776

[ref75] Velmans M. (2012). Reflexive monism psychophysical relations among mind, matter, and consciousness. J. Conscious. Stud. 19, 143–165.

[ref76] Von Glasersfeld E. (1987). “Learning as a constructive activity” in Problems of representation in the teaching and learning of mathematics (Mahwah, NJ, USA: Lawrence Erlbaum Associates), 3–17.

[ref77] Wiese W. Friston K. J. (2021). The neural correlates of consciousness under the free energy principle: from computational correlates to computational explanation. Philos. Mind Sci. 2:9. doi: 10.33735/phimisci.2021.81

[ref78] Woolf N. J. Hameroff S. R. (2001). A quantum approach to visual consciousness. Trends Cogn. Sci. 5, 472–478. doi: 10.1016/S1364-6613(00)01774-5, PMID: 11684479

[ref79] Yeung N. Summerfield C. (2012). Metacognition in human decision-making: confidence and error monitoring. Philos. Trans. R. Soc. B Biol. Sci. 367, 1310–1321. doi: 10.1098/rstb.2011.0416, PMID: 22492749 PMC3318764

[ref80] Yudkowsky E. (2008). “Artificial intelligence as a positive and negative factor in global risk” in Global Catastrophic Risks. eds. Bostrom N. Ćirkovic M. M., 308–345.

[ref81] Yudkowsky E. (2015). Rationality-from AI to zombies. Berkeley, CA, USA: Machine Intelligence Research Institute.

[ref82] Zeki S. Marini L. (1998). Three cortical stages of colour processing in the human brain. Brain J. Neurol. 121, 1669–1685. doi: 10.1093/brain/121.9.1669, PMID: 9762956

[ref83] Zhang B. Anderljung M. Kahn L. Dreksler N. Horowitz M. C. Dafoe A. (2021). Ethics and governance of artificial intelligence: evidence from a survey of machine learning researchers. J. Artif. Intell. Res. 71, 591–666. doi: 10.1613/jair.1.12895

